# White matter hyperintensities and risk of levodopa‐induced dyskinesia in Parkinson’s disease

**DOI:** 10.1002/acn3.50991

**Published:** 2020-02-07

**Authors:** Seok Jong Chung, Han Soo Yoo, Yang Hyun Lee, Jin Ho Jung, KyoungWon Baik, Byoung Seok Ye, Young H. Sohn, Phil Hyu Lee

**Affiliations:** ^1^ Department of Neurology Yonsei University College of Medicine Seoul South Korea; ^2^ Department of Neurology Yongin Severance Hospital Yonsei University Health System Yongin South Korea; ^3^ Severance Biomedical Science Institute Yonsei University College of Medicine Seoul South Korea

## Abstract

**Objective:**

To investigate whether the burden of white matter hyperintensities (WMHs) is associated with the risk of developing levodopa‐induced dyskinesia (LID) in Parkinson’s disease (PD).

**Methods:**

According to the Clinical Research Center for Dementia of South Korea WMH visual rating scale, 336 patients with drug‐naïve early stage PD (follow‐up >3 years) were divided into two groups of PD with minimal WMHs (PD‐WMH–; *n* = 227) and moderate‐to‐severe WMHs (PD‐WMH+; *n* = 109). The Cox regression model was used to estimate the hazard ratio for the development of LID in the PD‐WMH + group compared with the PD‐WMH– group, while adjusting for age at PD onset, sex, striatal dopamine depletion, and PD medication dose. Additionally, we assessed the effects of WMH burden rated by the Scheltens scale and regional WMH distribution on the development of LID.

**Results:**

Patients in the PD‐WMH + group were older and had more severe parkinsonian motor signs despite comparable striatal dopamine transporter availability than those in the PD‐WMH– group. Patients in the PD‐WMH + group had a higher risk of developing LID (hazard ratio, 2.66; *P* < 0.001) than those in the PD‐WMH– group after adjustment for other confounding factors. A greater WMH burden was associated with earlier occurrence of LID (hazard ratio, 1.04; *P* = 0.001), although the effects of WMHs on LID development did not exhibit region‐specific patterns.

**Interpretation:**

The present study demonstrates that the burden of WMHs is associated with occurrence of LID in patients with PD, suggesting comorbid WMHs as a risk factor for LID.

## Introduction

Levodopa‐induced dyskinesia (LID) occurs within several years in most patients with Parkinson’s disease (PD) who have undergone chronic dopamine replacement therapy,^1^ although the timing of the development of LID varies among patients. The pathogenesis underlying LID consists of two major events^2^, ^3^: loss of nigral dopaminergic neurons with an impaired capacity for striatal dopamine storage and plastic alterations of the basal ganglia structures in response to pulsatile stimulations with exogenous dopamine. Additionally, it has been widely gaining acceptance that both presynaptic and postsynaptic mechanisms are likely to result in aberrant plasticity in motor networks (i.e., striato‐cortical, cortico‐cortical, and cerebello‐cortical connections),^4^ which is also associated with the early development of LID.^5^, ^6^, ^7^

White matter hyperintensities (WMHs) are commonly observed in approximately 30–50% of patients with PD,^8^, ^9^ and several studies have demonstrated that a greater WMH burden is associated with severe motor impairments, especially axial motor symptoms, in PD.^10^, ^11^, ^12^ However, these previous studies were essentially cross‐sectional, and the effects of WMHs on long‐term motor outcomes, including the development of LID, have not yet been reported. Given that WM alterations may disconnect the motor cortex from the basal ganglia and cerebellum^13^ and even exert actions outside the visible lesions by remote effects,^14^, ^15^ we hypothesized that an increased WMH burden would be associated with a higher risk of LID in patients with PD. To test this, we investigated the occurrence of LID in 336 patients with de novo PD according to the severity of WMHs during a follow‐up period of approximately 5.5 years. In addition, we evaluated whether the impact of WMHs on the development of LID would depend on regional WMH distribution.

## Methods

### Subjects

We retrospectively reviewed the medical records of 484 consecutive patients with drug‐naïve early stage PD who visited the movement disorders outpatient clinic at Severance Hospital between April 2009 and September 2015. Of these, 148 patients were lost to follow‐up within 3 years and were excluded from the study. A total of 336 patients were treated with PD medications for at least 3 years, with doses adjusted for effective symptom control according to the patient’s response. PD was diagnosed according to the clinical diagnostic criteria of the United Kingdom Parkinson’s disease Society Brain Bank. All subjects underwent brain magnetic resonance imaging (MRI) scans at initial assessment, including fluid‐attenuated inversion recovery (FLAIR) sequence images, and ^18^F‐*N*‐(3‐fluoropropyl)‐2β‐carbon ethoxy‐3β‐(4‐iodophenyl) nortropane (^18^F‐FP‐CIT) positron emission tomography (PET). Parkinsonian motor symptoms were assessed using the Unified Parkinson’s Disease Rating Scale Part III (UPDRS‐III). PD medication doses were calculated as levodopa‐equivalent doses (LEDs).^16^ This study was approved by the Yonsei University Severance Hospital institutional review board, and the need for informed consent was waived because of the retrospective nature of the study.

### Rating of the WMH burden in patients with PD

#### Acquisition of FLAIR sequence images

Of the 336 patients with PD, 277 (82.4%) patients underwent brain MRI scans at Severance Hospital using a 3.0 T scanner (Achieva; Philips Medical System, Best, The Netherlands). The FLAIR sequence images were acquired with the following parameters: matrix, 512 × 512; slice number, 22; pixel spacing, 0.449 × 0.449 mm^2^; slice thickness, 5 mm; gap, 2 mm; field of view, 230 mm; repetition time, 11,000 msec; echo time, 125 msec; inversion time, 2,800 msec; flip angle, 90°. The other 59 (17.6%) patients underwent brain MRI scans including FLAIR sequence images at other hospitals before being referred to our hospital.

#### Classification of patients with PD according to the severity of WMHs

**Figure 1:**
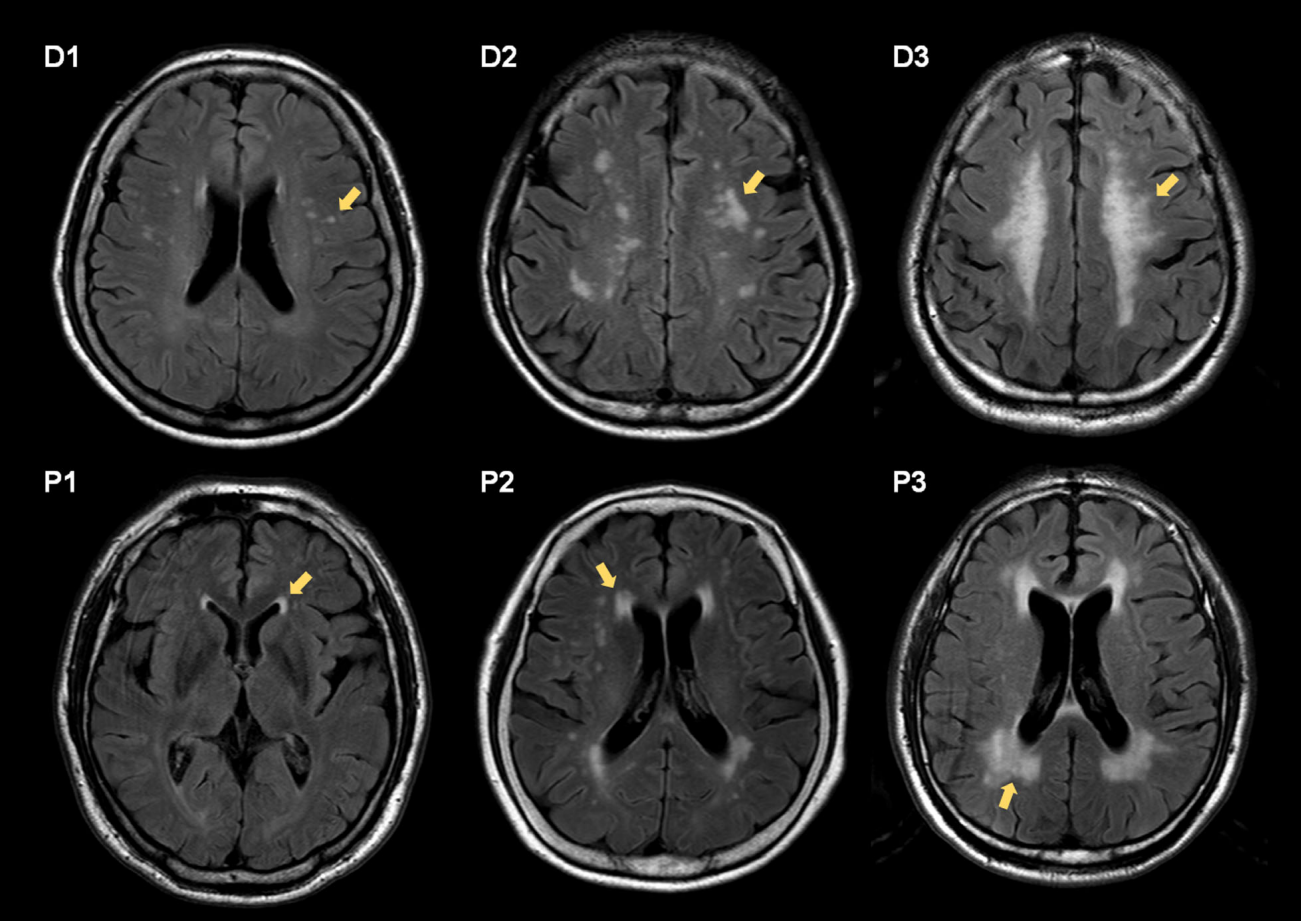
Examples of cases rated as P1, P2, P3, and D1, D2, D3.

The WMH burden of all 336 patients with de novo PD was rated on FLAIR images using the Clinical Research Center for Dementia of South Korea (CREDOS) WMH visual rating scale, which correlated well with the automatically measured WMH volume in a previous work.^17^, ^18^ First, the modified Fazekas scale was used to describe the extent of periventricular and deep WMHs.^19^ Hyperintensities evident in the axial slice just above the top of the lateral ventricles were considered to be periventricular WMH, whereas hyperintensities evident in the second or more axial slices above the top of the lateral ventricles were considered to be deep WMH.^17^ Periventricular WMHs were classified as P1 (cap and band <5 mm), P2 (between P1 and P3), or P3 (10 mm ≤cap or band) based on the size of cap and band, which were perpendicular and horizontal to the ventricle, respectively. Deep WMH were classified as D1 (maximum diameter of deep white matter lesion <10 mm), D2 (10 mm ≤lesion <25 mm), or D3 (lesion ≥25 mm) based on the longest diameter of the white matter lesions (see Fig. 1). Then, we divided the patients into two groups according to the CREDOS ischemia classification system: a PD group with minimal WMHs (D1P1 and D1P2; PD‐WMH–; *n* = 227) and a PD group with moderate‐to‐severe WMHs (D1P3, D2P1, D2P2, D2P3, D3P1, D3P2, and D3P3; PD‐WMH+, *n* = 109). WMH ratings were performed by two neurologists (LYH and YHS) blinded to the clinical information. The inter‐rater reliability for ratings of periventricular WMH and deep WMH was excellent (*κ* = 0.887 and 0.899, respectively), and the raters reached a consensus in cases of discrepancy.

#### A semi‐quantitative rating of WMHs using the Scheltens scale

Additionally, two neurologists (LYH and YHS) assessed the WMH burden of each patient using the Scheltens scale in which periventricular and lobar (frontal, parietal, temporal, and occipital) WMHs, as well as basal ganglia and infratentorial signal hyperintensities were rated separately in a semi‐quantitative manner.^20^ A final consensus rating between the two raters was used for the analysis.

### Quantitative analyses of the ^18^F‐FP‐CIT PET images

We used the same methodology to obtain and analyze the ^18^F‐FP‐CIT PET data to calculate the dopamine transporter (DAT) availability in the posterior putamen as previously described (see Data S1).^21^

### Assessment of the development of LID in the PD groups

After the diagnosis of PD, patients visited our outpatient clinic at 3‐ to 4‐month intervals, and two movement disorder specialists (SYH and LPH) assessed the presence of LID at every visit (average number of visits, 20.20 ± 6.37 times). The time from treatment initiation to the onset of LID was assessed with Kaplan–Meier estimates in the 109 patients with moderate‐to‐severe WMHs and the 227 patients with minimal WMHs. A log‐rank test was used to compare the Kaplan–Meier plots between the PD groups. To assess the effect of WMHs on the development of LID, the Cox regression model was used to estimate hazard ratios (HR) and 95% confidence intervals (CI) while adjusting for age at PD onset, sex, DAT availability in the posterior putamen, and LED per body weight at LID onset in patients with dyskinesia or at the last visit to the outpatient clinic in those without dyskinesia.

Additionally, to reduce the effects of potential confounding factors and provide the covariate balance, propensity scores were used to match the PD‐WMH + group with a subset of the PD‐WMH– group. The propensity score for the predicted probability of the severity of WMHs in each patient was estimated using a logistic regression model including the patient’s age at parkinsonian symptom onset, sex, PD duration, UPDRS‐III scores, and DAT availability in the posterior putamen as variables. Then, we created a propensity score‐matched cohort by matching each subject with moderate‐to‐severe WMHs to one subject with minimal WMHs (a 1:1 match). The propensity score matching was performed with the R software package, version 3.4.0 (http://www.r-project.org). Sensitivity analyses were then performed in these matched samples (i.e., the 109 patients in the PD‐WMH + group and the matched 109 patients in the PD‐WMH– group; see Data S1).

### Assessment of the development of LID according to the regional WMH burden

Cox regression analysis was performed to assess the effect of the regional WMH burden rated by the Scheltens scale^20^ on the development of LID, while adjusting for age at PD onset, sex, DAT availability in the posterior putamen, and LED per body weight. In addition, to demonstrate which regional WMHs (i.e., periventricular, lobar [frontal, parietal, temporal, and occipital], basal ganglia, and infratentorial WMHs) is more explanatory and informative in predicting the development of LID, the following parameters were calculated for each Cox regression model: the Akaike information criterion (AIC), the discriminatory ability assessed by the linear trend chi‐squared test, the concordance index (Harrell’s C‐index), and a global concordance probability (integrated area under the curve [iAUC]). A smaller AIC and larger discriminatory ability, Harrell’s C‐index, and iAUC indicate the preferred model with better predictive accuracy. We also compared the iAUC between the Cox regression models using a bootstrapping method with resampling 1000 times.

### Statistical analyses

The baseline demographic characteristics, WMHs rated by the Scheltens scale, and the striatal DAT availability of the PD groups were compared with Student’s *t*‐tests and Pearson’s chi‐squared tests. The effect of WMHs on the development of LID was assessed with a log‐rank test and the Cox regression model as described above. Pearson’s correlation coefficients were calculated to assess the correlation between the WMH rating scores in each brain region. The statistical analyses were performed with SPSS (version 23.0; IBM Corporation, Armonk, NY, USA), and results with a two‐tailed p‐value < 0.05 were considered statistically significant.

## Results

### Baseline clinical characteristics of the patients with PD

Baseline demographic characteristic and clinical features are listed in Table 1. The patients in the PD‐WMH + group (*n* = 109) were older and had higher UPDRS‐III scores and a higher prevalence of hypertension and diabetes mellitus than those in the PD‐WMH– group (*n* = 227). There were no significant differences in sex, PD duration, and motor subtypes between the PD groups. The PD‐WMH + group showed a greater WMH burden, as rated by the Scheltens scale^20^, in the periventricular, lobar, basal ganglia, and infratentorial regions than the PD‐WMH– group. DAT availability in the posterior putamen was comparable between the PD‐WMH + group (1.31 ± 0.45) and PD‐WMH– group (1.35 ± 0.42; *P* = 0.181).

**Table 1:** Baseline demographic characteristics in patients with PD.

	Overall series			Propensity score‐matched pairs^a^		
	PD‐WMH‐ (*n* = 227)	PD‐WMH+ (*n* = 109)	*P*‐value	PD‐WMH‐ (*n* = 109)	PD‐WMH+ (*n* = 109)	*P*‐value
Demographic characteristics						
Age (years)	63.92 ± 9.59	70.14 ± 7.82	<0.001	69.49 ± 8.39	70.14 ± 7.82	0.553
Female, No. (%)	113 (49.5%)	56 (51.4%)	0.784	51 (46.8%)	56 (51.4%)	0.498
Onset of age (years)	62.45 ± 9.74	68.68 ± 7.85	<0.001	68.08 ± 8.46	68.68 ± 7.85	0.590
PD duration (months)	17.43 ± 14.71	17.60 ± 16.23	0.926	16.88 ± 14.18	17.60 ± 16.23	0.729
UPDRS‐III	21.45 ± 9.06	26.30 ± 10.12	<0.001	24.17 ± 8.73	26.30 ± 10.12	0.098
Vascular risk factors						
Hypertension	74 (32.6%)	58 (53.2%)	<0.001	45 (41.3%)	58 (53.2%)	0.078
Diabetes mellitus	28 (12.3%)	26 (23.9%)	0.007	17 (15.6%)	26 (23.9%)	0.126
Dyslipidemia	32 (14.1%)	22 (20.2%)	0.155	17 (15.6%)	22 (20.2%)	0.377
Body mass index	23.52 ± 2.99	23.17 ± 3.25	0.334	23.52 ± 3.04	23.17 ± 3.25	0.410
LID occurrence, No. (%)	58 (25.6%)	43 (39.4%)	0.009	24 (22.0%)	43 (39.4%)	0.005
WMH burden^b^						
Periventricular WMHs	1.77 ± 1.12	4.27 ± 1.27	<0.001	2.08 ± 1.19	4.27 ± 1.27	<0.001
Lobar WMHs	3.89 ± 3.01	12.13 ± 3.78	<0.001	4.45 ± 3.24	12.13 ± 3.78	<0.001
Basal ganglia WMHs	0.48 ± 0.98	2.84 ± 2.94	<0.001	0.61 ± 1.11	2.84 ± 2.94	<0.001
Infratentorial WMHs	0.36 ± 0.83	0.95 ± 1.33	<0.001	0.43 ± 0.97	0.95 ± 1.33	<0.001
Total WMHs	6.40 ± 4.16	20.06 ± 6.79	<0.001	7.50 ± 4.45	20.06 ± 6.79	<0.001
DAT availability						
Posterior putamen	1.35 ± 0.42	1.31 ± 0.45	0.181	1.27 ± 0.38	1.31 ± 0.45	0.536

Values are expressed as mean ± standard deviation or number (percentage). Abbreviations: PD, Parkinson’s disease; PD‐WMH‐, PD group with minimal white matter hyperintensities (WMHs); PD‐WMH+, PD group with moderate‐to‐severe WMHs; UPDRS‐III, the Unified Parkinson’s Disease Rating Scale Part III; LID, levodopa‐induced dyskinesia; DAT, dopamine transporter.

a Propensity score matching using a logistic regression model including the age at onset, sex, PD duration, UPDRS‐III scores, and DAT availability in the posterior putamen as predictors.

b Based on the Scheltens scale (Journal of the Neurological Sciences 1993;114:7–12).

Propensity score subsamples (109 in the PD‐WMH + group and 109 matched samples in the PD‐WMH– group) revealed no significant differences in baseline demographic characteristics and striatal DAT availability between the PD groups, although the PD‐WMH + group tended to have higher UPDRS‐III scores (*P* = 0.098) and a higher prevalence of hypertension (*P* = 0.078) than the matched PD‐WMH– group. The PD‐WMH + group exhibited a greater WMH burden based on the Scheltens scale^20^ than the matched PD‐WMH– group (Table 1).

### Development of LID in the PD groups according to WMH severity

**Figure 2:**
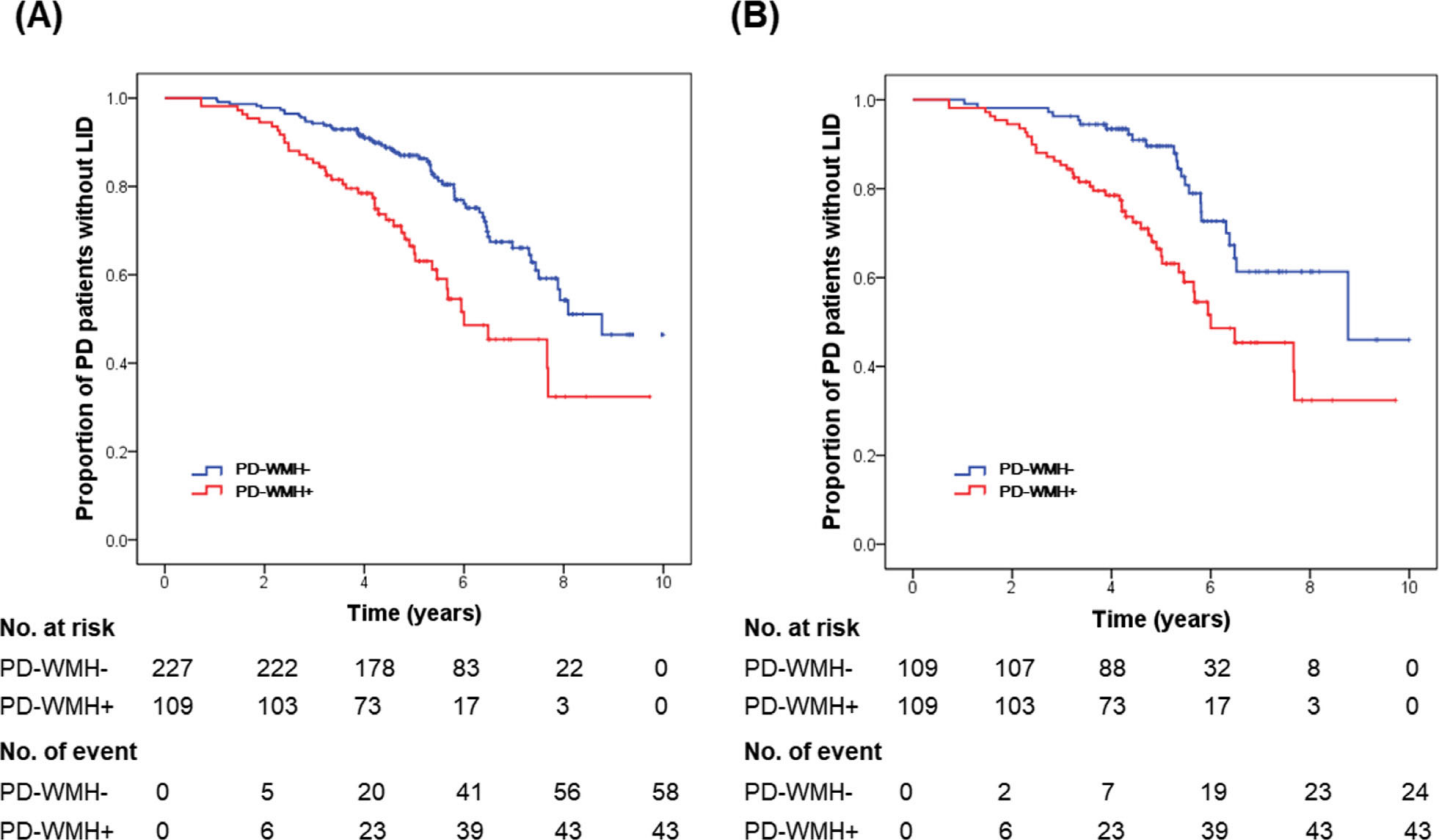
Curves of Kaplan–Meier estimates of the onset of levodopa‐induced dyskinesia (LID) after treatment initiation in patients with moderate‐to‐severe WMHs and matched patients with minimal WMHs. (A) Overall series. (B) Propensity score‐matched pairs. The PD‐WMH + group had a higher risk of early development of LID than the PD‐WMH– group (*P*_log‐rank_ < 0.001). The crosses in the graphs indicate censored data. Abbreviations: PD‐WMH–, PD group with minimal white matter hyperintensities; PD‐WMH+, PD group with moderate‐to‐severe white matter hyperintensities.

During the follow‐up period, LID developed in 43 (39.4%) of the 109 patients in the PD‐WMH + group (follow‐up duration, 5.43 ± 1.66 years) and in 58 (25.6%) of the 227 patients in the PD‐WMH– group (follow‐up duration, 5.99 ± 1.87 years). The Kaplan–Meier analyses revealed that the PD‐WMH + group had a higher risk of developing LID than the PD‐WMH– group (*P*_log‐rank_ <0.001; Fig. 2A). The HR for developing LID after starting PD medication in the PD‐WMH + group compared with the PD‐WMH– group was 2.660 (95% CI [1.742 − 4.062]; *p* < 0.001), indicating that the PD‐WMH + group had a higher risk of developing LID than the PD‐WMH– group when the model was adjusted for age at PD onset, sex, baseline striatal dopamine depletion, and LED per body weight. In addition, older age at onset, male sex, and mildly decreased DAT availability in the posterior putamen were associated with a lower risk of developing LID (Table 2).

**Table 2:** Cox regression analysis for the development of levodopa‐induced dyskinesia in Parkinson’s disease groups according to white matter hyperintensities.

Factors	Overall series		Propensity score‐matched pairs^a^	
	Hazard ratio (95% CI)	*P*‐value	Hazard ratio (95% CI)	*P*‐value
Group (PD‐WMH + vs. PD‐WMH‐)	2.660 (1.742−4.062)	<0.001	2.429 (1.459–4.042)	0.001
Age at PD onset	0.973 (0.953–0.994)	0.014	0.979 (0.951–1.008)	0.149
Sex (Female vs. Male)	1.718 (1.143–2.581)	0.009	1.886 (1.144–3.109)	0.013
DAT availability in posterior putamen	0.592 (0.373–0.941)	0.027	0.583 (0.328–1.036)	0.066
LED per body weight	1.005 (0.982–1.028)	0.670	1.017 (0.963–1.074)	0.547

Abbreviations: PD, Parkinson’s disease; PD‐WMH+, PD group with moderate‐to‐severe white matter hyperintensities; PD‐WMH‐, PD group with minimal white matter hyperintensities; DAT, dopamine transporter; LED, levodopa‐equivalent; CI, confidence interval.

a Propensity score matching using a logistic regression model including the age at onset, sex, PD duration, UPDRS‐III scores, and DAT availability in the posterior putamen as predictors.

We also obtained consistent results in propensity score subsamples: The PD‐WMH + group had a higher risk of developing LID than the matched PD‐WMH– group (*P*_log‐rank_ <0.001, Fig. 2B; HR 2.429, 95% CI [1.459 − 4.042], *P* = 0.001, Table 2).

### Development of LID according to the regional WMH burden

The Cox regression model demonstrated that a greater total WMH burden based on the Scheltens scale was associated with a higher risk of developing LID in either overall samples (HR, 1.044; 95% CI, [1.018 − 1.071]; *P* = 0.001) or propensity score subsamples (HR, 1.040; 95% CI, [1.012 − 1.069]; *P* = 0.005). Younger age at onset, female sex, and severely decreased DAT availability in the posterior putamen were also associated with a higher risk of developing LID (Table 3).

**Table 3:** Cox regression analysis for the development of levodopa‐induced dyskinesia according to white matter hyperintensities assessed by the Scheltens scale.

Factors	Overall series		Propensity score‐matched pairs^a^	
	Hazard ratio (95% CI)	*P*‐value	Hazard ratio (95% CI)	*P*‐value
Total WMHs	1.044 (1.018–1.071)	0.001	1.040 (1.012–1.069)	0.005
Age at PD onset	0.971 (0.949–0.994)	0.013	0.968 (0.940–0.998)	0.037
Sex (Female vs. Male)	1.594 (1.060–2.396)	0.025	1.718 (1.042–2.830)	0.034
DAT availability in posterior putamen	0.545 (0.336–0.882)	0.014	0.548 (0.300–1.001)	0.050
LED per body weight	1.006 (0.985–1.028)	0.566	1.030 (0.977–1.087)	0.273

Abbreviations: WMHs, white matter hyperintensities; PD, Parkinson’s disease; DAT, dopamine transporter; LED, levodopa‐equivalent; CI, confidence interval.

a Propensity score matching using a logistic regression model including the age at onset, sex, PD duration, UPDRS‐III scores, and DAT availability in the posterior putamen as predictors.

Moreover, we investigated whether the impact of WMHs on the development of LID would depend on the regional WMH distribution. The Cox regression models demonstrated that periventricular (HR, 1.139; 95% CI [0.998 − 1.300]; *P* = 0.053), lobar (HR, 1.067; 95% CI [1.025 − 1.110]; *P* = 0.002), basal ganglia (HR, 1.132; 95% CI [1.034 − 1.239]; *P* = 0.007), and infratentorial (HR, 1.216; 95% CI [1.022 − 1.445]; *P* = 0.027) WMHs were significantly associated with LID occurrence (Table S1). In addition, WMH burden in the frontal (HR, 1.196; 95% CI [1.047 − 1.366]; *P* = 0.008), parietal (HR, 1.214; 95% CI [1.073 − 1.373]; *P* = 0.002), temporal (HR, 1.187; 95% CI [1.026 − 1.372]; *P* = 0.021), and occipital (HR, 1.261; 95% CI [1.081 − 1.470]; *P* = 0.003) lobes significantly affected the occurrence of LID (Table S2). The predictive ability of each Cox regression model was comparable in terms of the AIC, discriminatory ability, and Harrell’s C‐index (Table 4). In particular, there were no significant differences in the iAUC between the Cox regression models, which were calculated using a bootstrapping method with resampling 1000 times. In correlation analyses, there was a strong correlation between WMH rating scores in each brain region with a sufficiently large effect size (correlation coefficients >0.5; Table S3).

**Table 4:** The predictive accuracy of the Cox regression models according to white matter hyperintensities of each brain region.

	AIC	Linear trend *χ* ^ 2^	Harrell’s C	iAUC
Total WMH	1021.305	23.85	0.655	0.636
Periventricular WMH	1027.626	17.59	0.647	0.617
Lobar WMH	1021.858	23.35	0.655	0.634
Frontal WMH	1024.405	20.98	0.645	0.601
Parietal WMH	1022.108	22.69	0.656	0.635
Temporal WMH	1026.333	18.91	0.633	0.620
Occipital WMH	1023.419	21.40	0.643	0.628
Basal ganglia WMH	1025.168	19.94	0.637	0.622
Infratentorial WMH	1027.006	18.25	0.631	0.617

The Akaike information criterion (AIC) was calculated for each Cox regression model to demonstrate which regional WMHs is more explanatory and informative in predicting the development of LID (a smaller AIC indicates the preferred model). Additionally, discriminatory ability (linear trend *χ* ^2^ test), the concordance index (Harrell’s C‐index), and a global concordance probability (integrated area under the curve [iAUC]) were also calculated for each Cox regression model to assess the predictive accuracy (larger discriminatory ability, Harrell’s C‐index, and iAUC indicate better predictive ability). There were no significant differences in iAUC between the Cox regression models, which were calculated using a bootstrapping method with resampling 1000 times, suggesting that the predictive accuracy of each Cox regression model was comparable.

## Discussion

This study investigated the effects of baseline WMH burden on the development of LID in patients with drug‐naïve early stage PD. The major findings were as follows: (1) patients with PD with moderate‐to‐severe WMHs were older and had higher UPDRS‐III scores than those with minimal WMHs. (2) Patients in the PD group with moderate‐to‐severe WMHs had a higher risk of developing LID than those in the PD group with minimal WMHs, after adjustment for confounding factors such as age at onset, sex, striatal DAT availability, and doses of PD medications. (3) A greater WMH burden was associated with an earlier occurrence of LID, although the effects of WMHs on the development of LID were not regionally dependent. These findings suggest that baseline WMHs can act as a predictive marker or therapeutic target for the development of LID in patients with early stage PD.

The underlying mechanisms of LID have traditionally been described at the presynaptic and postsynaptic levels.^2^, ^3^ Presynaptic mechanisms refer to an impaired regulation of the striatal synaptic dopamine level as a consequence of the loss of nigral dopaminergic neurons and hyperinnervation of serotonergic fibers.^22^ Postsynaptically, intermittent dopamine receptor activation results in plastic alterations in gene expression and neuropeptide formation within the striatal pathways,^23^ which subsequently lead to aberrant firing patterns of basal ganglia output neurons. Taking the pathogenesis of LID into consideration, age at PD onset, disease severity, and regimen of chronic dopamine replacement therapy are obvious risk factors for developing LID.^24^ Importantly, there is increasing evidence that patients with PD with LID exhibit an impaired plasticity within the larger motor network^4^ (i.e., absent or poor plastic response in the primary motor cortex [M1],^5^ erroneous cortico‐striatal representations,^25^ impaired cerebello‐cortical connections,^26^ and abnormal inhibitory regulation in the cortico‐cortical connections^27^). Polymorphism of the brain‐derived neurotrophic factor gene, which regulates the synaptic plasticity and efficacy in the cortex and striatum, has been also reported to influence the time to develop LID.^6^ Several studies have shown that neuromodulation of the aberrant motor maps has potential therapeutic implications for LID.^26^, ^27^

Ample evidence has suggested that comorbid WMHs could negatively influence the motor features^8^, ^10^ and poor levodopa response^11^, ^28^ in PD. Likewise, patients in the PD‐WMH + group in this study showed more severe motor deficits than those in the PD‐WMH– group despite the comparable striatal DAT availability between the groups at an initial assessment. As a possible explanation for the clinical impact of WMHs in PD, WM alterations may interrupt the subcortical motor circuits,^13^ leading to more severe parkinsonian motor deficits.^29^ Furthermore, WM lesions outside the frontal regions, where major tracts linked to higher‐order motor control are located, also appear to disconnect the motor cortex from the basal ganglia and cerebellum by either perilesional or remote effects.^14^, ^15^ Additionally, various pathologies related to WMHs, including vascular pathology,^30^ low‐grade inflammation,^31^ Wallerian degeneration,^32^ and axonal transport disruption,^33^ may facilitate the neurodegenerative process in patients with PD with a greater extra‐nigral pathological burden.^34^ In the present study, we provide evidence for the first time that a severe WMH burden was associated with the early development of LID, even after adjusting for confounding factors such as LED and nigrostriatal dopamine depletion. Accordingly, based on previous and current studies, the WMH burden seems to be an important clinical parameter, acting as a predictor of parkinsonian motor outcome, as well as LID development in patients with PD.

In this study, the risk of developing LID did not differ according to the regional WMH distribution. Indeed, several previous studies have emphasized the frontal area, which is structurally and functionally interconnected to the striatum, as the core neural correlates of LID. In neuroimaging studies, Cerasa and colleagues have consistently reported that the right inferior frontal cortex would play a key role in the pathophysiology of LID, with an increase in gray matter volume or thickness^35^, ^36^ and an abnormal pattern of functional connectivity in this region.^27^, ^37^ Recently, we found an association between the development of LID and frontal lobe‐based cognitive decline,^38^ suggesting that abnormal striatal and cortical plasticity in the frontal area might result in both aberrant motor and cognitive loops. In this study, the lack of regional predominance in the impact of WMHs on LID development might be due to a strong correlation between the WMH rating scores in each brain region. In particular, WMH rating scores of the frontal lobe were well correlated with those of the other lobes (parietal lobe, *γ* = 0.754; temporal lobe, *γ* = 0.654; occipital lobe, *γ* = 0.577) with a sufficiently large effect size. The total WMH severity was also well correlated with the subregional WMH severity (periventricular, *γ* = 0.836; lobar, *γ* = 0.717), suggesting that the pathological burden related to WMHs would affect the entire brain without region‐specific predominant patterns. Additionally, the remote effects of WM lesions^14^, ^15^ might make it possible to interrupt the subcortical motor circuits even if the frontal area was not involved. Further studies using diffusion tensor imaging, which can detect microstructural WM alterations (e.g., structural WM connectivity) early,^39^ would provide more answers with respect to the conclusions of this study.

Our study has some limitations. First, the age at PD onset and baseline motor severity, which are important risk factors for developing LID,^24^ differed between the PD groups. However, consistent results were obtained from the sensitivity analyses using the propensity score‐matched samples. Furthermore, the PD‐WMH + group had a higher risk of developing LID despite their older age at PD onset. Individual variability in the regimens of dopamine replacement therapy may also affect the development of LID,^40^ although the LED per body weight at LID onset or last visit was included as a covariate in the Cox regression model. Second, the severity of WMHs was not quantified as the WMH volume but rather visually assessed based on the CREDOS ischemia classification system^17^ and the Scheltens scale.^20^ However, these visual rating scales are known to correlate well with the automatically measured WMH volume.^17^ Third, the burden of vascular risk factors, which might affect the progression of WMHs, could have variably changed during follow‐up; however, this confounding effect is difficult to assess in a retrospective study design. Finally, minimal to mild LID may not be detected owing to our determination of the presence of LID via medical records. In addition, there is a lack of detailed information on the adverse effects of LID development on quality of life.

In conclusion, this study demonstrates that the burden of WMHs is associated with the occurrence of LID in patients with PD. These findings suggest that comorbid WMHs may affect the subcortical motor pathways, leading to an increased risk of developing LID.

## Conflict of Interest

The authors report that they do not have any conflicts of interest.

## Author Contributions

S.J.C. and P.H.L conceptualized and designed the study. S.J.C., H.S.Y., Y.H.L., J.H.J., K.W.B., B.S.Y., Y.H.S., and P.H.L acquired and analyzed the data. S.J.C., Y.H.S., and P.H.L drafted the significant portion of the manuscript or figures.

## Supporting information


**Data S1** **.** Supplementary Methods.
**Table S1** **.** Cox regression analysis for the development of levodopa‐induced dyskinesia according to regional white matter hyperintensities assessed by the Scheltens scale.
**Table S2** **.** Cox regression analysis for the development of levodopa‐induced dyskinesia according to lobar white matter hyperintensities.
**Table S3** **.** Correlation coefficients between the white matter hyperintensities of each brain region.
