# Supplementary material for: White matter hyperintensities and risk of levodopa‐induced dyskinesia in Parkinson’s disease
Source: Ann Clin Transl Neurol. 2020 Feb 7;7(2):229–38. doi: 10.1002/acn3.50991 (PMC7034502; doi:10.1002/acn3.50991)
Supplement: Supplementary file 1 — Data S1 . Supplementary Methods. Table S1 . Cox regression analysis for the development of levodopa‐induced dyskinesia according to regional white matter hyperintensities assessed by the Scheltens scale. Table S2 . Cox regression analysis for the development of levodopa‐induced dyskinesia according to lobar white matter hyperintensities. Table S3 . Correlation coefficients between the white matter hyperintensities of each brain region. [file ACN3-7-229-s001.docx]

**Supplementary Methods**

**^18^F-FP-CIT PET acquisition**

The ^18^F-FP-CIT PET scans were acquired using a GE Discovery STe PET-CT scanner (GE Healthcare, Milwaukee, WI, USA), which obtains images with three-dimensional resolution of 2.3-mm full width at half maximum. After the subjects fasted for at least 6 h, they were intravenously injected with 5mCi (185 MBq) of ^18^F-FP-CIT. 90 min after the injection, PET images were acquired for 20 min in the three-dimensional mode, and spiral CT images were obtained for attenuation correction with 120 kVp and 200 mA.

**Quantitative analyses of the ^18^F-FP-CIT PET images**

Image processing was performed using SPM8 (Wellcome Department of Imaging Neuroscience, Institute of Neurology, University College London, London, UK) with MATLAB 2013a for Windows (Math Works, Natick, MA, USA). All reconstructed PET images were spatially normalized to the Montreal Neurology Institute template using a standard ^18^F-FP-CIT PET template that was generated from ^18^F-FP-CIT PET and T1 MRI of 13 healthy controls. The volumes of interest (VOI) for the bilateral posterior putamen and one occipital VOI (i.e., calcarine fissure and surrounding cortex [V1]^1^) were drawn on a co-registered spatially normalized single T1 MRI and ^18^F-FP-CIT PET template image on MRIcro version 1.37 (Chris Rorden, Columbia, SC, USA). The boundaries of the posterior putamen were defined as described in our previous work^2^ and were adjusted by a minor translation in our in-house editing software ANIQUE.^3^ Dopamine transporter (DAT) availability was calculated by the non-displaceable binding potential, which was defined as (mean standardized uptake value of the striatal sub-regions VOI–mean standardized uptake value of the occipital VOI)/(mean standardized uptake of the occipital VOI).

**Propensity score matching of the patients according to WMH**

To reduce the effects of potential confounding factors and provide the covariate balance, propensity scores were used to match the PD-WMH+ group with a subset of the PD-WMH- group. The propensity score for the predicted probability of the severity of WMHs in each patient was estimated using a logistic regression model including the patient’s age at parkinsonian symptom onset, sex, PD duration, UPDRS-III scores, and DAT availability in the posterior putamen as variables. The propensity score represented a balancing score, and thus the distribution of the covariates would be the same between the groups if a set of subjects had the same propensity score.^4^ We created a propensity-score-matched cohort by matching each subject with moderate-to-severe WMHs to one subject with minimal WMHs (a 1:1 match). A nearest-neighbor-matching algorithm was used to match the patients: At each matching step, the PD-WMH- subject who was closest to the PD-WMH+ subject on the propensity score was selected. If multiple PD-WMH- subjects had propensity scores that were equally close to that of the PD-WMH+ subject, one of these PD-WMH- subjects was chosen at random.^4^ The propensity score matching was performed with the R software package, version 3.4.0 (http://www.r-project.org).

**References**

1. Tzourio-Mazoyer N, Landeau B, Papathanassiou D, et al. Automated anatomical labeling of activations in SPM using a macroscopic anatomical parcellation of the MNI MRI single-subject brain. Neuroimage 2002;15:273-289.

2. Chung SJ, Yoo HS, Oh JS, et al. Effect of striatal dopamine depletion on cognition in de novo Parkinson's disease. Parkinsonism Relat Disord 2018;51:43-48.

3. Oh JS, Oh M, Chung SJ, Kim JS. Cerebellum-specific 18F-FDG PET analysis for the detection of subregional glucose metabolism changes in spinocerebellar ataxia. Neuroreport 2014;25:1198-1202.

4. Austin PC. An Introduction to Propensity Score Methods for Reducing the Effects of Confounding in Observational Studies. Multivariate Behav Res 2011;46:399-424.

**Supplementary Table 1. Cox regression analysis for the development of levodopa-induced dyskinesia according to regional white matter hyperintensities assessed by the Scheltens scale**

|  | Periventricular | |  | Lobar | |  | Basal ganglia | |  | Infratentorial | |
| --- | --- | --- | --- | --- | --- | --- | --- | --- | --- | --- | --- |
| Factors | HR (95% CI) | *p*-value |  | HR (95% CI) | *p*-value |  | HR (95% CI) | *p*-value |  | HR (95% CI) | *p*-value |
| WMHs | 1.139 (0.998−1.300) | 0.053 |  | 1.067 (1.025−1.110) | 0.002 |  | 1.132 (1.034−1.239) | 0.007 |  | 1.216 (1.022−1.445) | 0.027 |
| Age at PD onset | 0.977 (0.954−1.000) | 0.045 |  | 0.973 (0.951−0.995) | 0.017 |  | 0.980 (0.960−1.001) | 0.066 |  | 0.984 (0.963−1.005) | 0.128 |
| Sex (Female vs. Male) | 1.587 (1.056−2.387) | 0.026 |  | 1.612 (1.073−2.422) | 0.022 |  | 1.655 (1.102−2.485) | 0.015 |  | 1.605 (1.068−2.412) | 0.023 |
| DAT availability | 0.549 (0.339−0.892) | 0.016 |  | 0.571 (0.352−0.925) | 0.023 |  | 0.508 (0.309−0.833) | 0.007 |  | 0.483 (0.295−0.789) | 0.004 |
| LED per body weight | 1.005 (0.984−1.027) | 0.632 |  | 1.007 (0.985−1.029) | 0.551 |  | 1.006 (0.986−1.027) | 0.570 |  | 1.001 (0.981−1.021) | 0.916 |

Abbreviations: WMHs, white matter hyperintensities; PD, Parkinson’s disease; DAT, dopamine transporter; LED, levodopa-equivalent; HR, hazard ratio; CI, confidence interval.

**Supplementary Table 2. Cox regression analysis for the development of levodopa-induced dyskinesia according to lobar white matter hyperintensities**

|  | Frontal | |  | Parietal | |  | Temporal | |  | Occipital | |
| --- | --- | --- | --- | --- | --- | --- | --- | --- | --- | --- | --- |
| Factors | HR (95% CI) | *p*-value |  | HR (95% CI) | *p*-value |  | HR (95% CI) | *p*-value |  | HR (95% CI) | *p*-value |
| WMHs | 1.196 (1.047−1.366) | 0.008 |  | 1.214 (1.073−1.373) | 0.002 |  | 1.187 (1.026−1.372) | 0.021 |  | 1.261 (1.081−1.470) | 0.003 |
| Age at PD onset | 0.978 (0.956−0.999) | 0.042 |  | 0.973 (0.951−0.995) | 0.017 |  | 0.980 (0.959−1.002) | 0.069 |  | 0.975 (0.954−0.997) | 0.027 |
| Sex (Female vs. Male) | 1.547 (1.027−2.331) | 0.037 |  | 1.635 (1.089−2.456) | 0.018 |  | 1.666 (1.111−2.500) | 0.014 |  | 1.677 (1.117−2.517) | 0.013 |
| DAT availability | 0.592 (0.363−0.964) | 0.035 |  | 0.553 (0.341−0.897) | 0.016 |  | 0.545 (0.335−0.887) | 0.015 |  | 0.541 (0.334−0.877) | 0.013 |
| LED per body weight | 1.006 (0.984−1.028) | 0.605 |  | 1.007 (0.985−1.029) | 0.548 |  | 1.005 (0.983−1.027) | 0.667 |  | 1.007 (0.986−1.027) | 0.526 |

Abbreviations: WMHs, white matter hyperintensities; PD, Parkinson’s disease; DAT, dopamine transporter; LED, levodopa-equivalent; HR, hazard ratio; CI, confidence interval.

**Supplementary Table 3. Correlation coefficients between the white matter hyperintensities of each brain region**

|  | Total | Periventricular | Lobar | Frontal | Parietal | Temporal | Occipital |
| --- | --- | --- | --- | --- | --- | --- | --- |
| Total | - | 0.836 | 0.937 | 0.844 | 0.847 | 0.761 | 0.760 |
| Periventricular | 0.836 | - | 0.758 | 0.694 | 0.702 | 0.587 | 0.605 |
| Lobar | 0.937 | 0.758 | - | 0.879 | 0.913 | 0.823 | 0.812 |
| Frontal | 0.844 | 0.694 | 0.879 | - | 0.754 | 0.654 | 0.577 |
| Parietal | 0.847 | 0.702 | 0.913 | 0.754 | - | 0.658 | 0.683 |
| Temporal | 0.761 | 0.587 | 0.823 | 0.654 | 0.658 | - | 0.568 |
| Occipital | 0.760 | 0.605 | 0.812 | 0.577 | 0.683 | 0.568 | - |
